# The motivational architecture of sexual orientation across development and evolutionary persistence

**DOI:** 10.3389/fpsyg.2026.1906479

**Published:** 2026-10-26

**Authors:** Andrea S. Camperio Ciani

**Affiliations:** Department of Philosophy, Sociology, Education and Applied Psychology (FISPPA), University of Padua, Padua, Italy

**Keywords:** androphilia, antagonistic pleiotropy, balancing selection, developmental systems, gynephilia, polygenic architecture, sexual orientation, sexually antagonistic selection

## Abstract

Understanding the persistence and development of human same-sex orientation may be clarified by distinguishing genetic architecture, evolutionary maintenance, developmental assembly, motivational organization, behavior, identity, and socioecological context without treating them as independent or linearly ordered causes. This conceptual analysis uses a targeted narrative synthesis of evolutionary, genetic, developmental, neurobiological, and socioecological evidence to examine a reciprocal multilevel framework centered on androphilic and gynephilic motivation as attraction-related dispositions that may stabilize or remain plastic through development. Family, twin, and genomic findings are broadly consistent with a partly heritable and highly polygenic contribution to sexual orientation. For male androphilia, some pedigree and fecundity findings are compatible with a specific X-linked sexually antagonistic selection (SAS) model, particularly where increased reproductive output extends to non-parental female relatives in the maternal lineage. Across populations and study designs, however, the evidence is heterogeneous, and recent work separating fraternal birth order from female fecundity cautions against treating maternal fecundity as a general proxy for SAS. The X-linked model is therefore considered a falsifiable submodel within a broader polygenic framework in which antagonistic pleiotropy may contribute to maintenance. For female same-sex orientation, stable lesbian orientation is distinguished from broader gynephilic motivation and sexual fluidity. Bilateral pedigree compensation is compatible with balancing selection but requires independent replication, whereas alloparenting and co-wife hypotheses address potentially context-dependent functions of female sexual fluidity. Developmental mechanisms—including maternal immune effects, prenatal endocrine variation, neural differentiation, childhood trajectories, and developmental stochasticity—may contribute to phenotypic realization rather than acting as alternatives to genetic liability. Motivational organization is proposed as an intermediate level linking developmental assembly to relatively stable orientation and context-sensitive behavioral and identity expression. Cross-cultural evidence further suggests that social institutions can modify expression, kin-directed behavior, identity, and realized fitness without constituting a universal maintenance mechanism. Overall, the reviewed evidence is consistent with a plural, phenotype-specific framework in which polygenic liability, developmental processes, motivational organization, and socioecological context interact reciprocally. Specific maintenance mechanisms, including X-linked SAS and balancing selection, remain open to empirical test. The framework generates predictions integrating genomic, pedigree, developmental, and socioecological measures within clearly defined phenotypes and populations.

## 1 Introduction

The central problem addressed is evolutionary: persistent same-sex sexual orientations can reduce direct reproductive output, yet same-sex attractions and orientations recur across cultures and historical periods, posing an apparent paradox for selection (Williams, 1966; Camperio Ciani et al., 2004, 2008).

Addressing this paradox benefits from distinguishing explanatory questions without treating them as causally isolated domains. Following Tinbergen (1963), ultimate accounts concern evolutionary history and the population-level maintenance of traits, whereas proximate and developmental accounts concern the mechanisms and ontogenetic processes through which phenotypes are assembled. In this article, this distinction is used as an analytical heuristic rather than as an ontological separation between independent causes. Developmental processes generate and modify the phenotypic variation on which selection acts, while evolutionary history constrains developmental systems; reciprocal organism–environment interactions can therefore connect explanatory levels across both ontogenetic and evolutionary time (Laland et al., 2011; Oyama, 2000; Griffiths and Tabery, 2013; West-Eberhard, 2003).

Accordingly, selection-based maintenance models make predictions about multigenerational signatures in fecundity and pedigrees, whereas developmental hypotheses address pathways through which attraction-related dispositions emerge and become relatively stable or remain plastic. These forms of explanation are complementary only when their empirical signatures are distinguished rather than assumed to be independent (Rice and Gavrilets, 2014; Blanchard, 2004; Bogaert, 2006). Genetic, hormonal, neural, behavioral, and socioecological influences are therefore treated as interacting components of developmental systems rather than as separable additive causes.

Comparative and cultural evidence further indicates that the expression and social embedding of same-sex orientations can vary substantially across contexts. Cultural institutions, mating systems, kinship structures, stigma, and socially recognized gender roles can alter the opportunities, costs, and meanings associated with same-sex attraction without implying that cultural context alone generates the underlying motivational phenotype (Vasey and VanderLaan, 2010; VanderLaan and Vasey, 2011; VanderLaan et al., 2012, 2017; Camperio Ciani et al., 2016a).

At the proximate and developmental level, prenatal endocrine variation, maternal immune processes associated with fraternal birth order, neural differentiation, childhood behavior, and continuing social experience may all contribute probabilistically to developmental trajectories (Hines, 2004, 2011; Balthazart, 2011; Bao and Swaab, 2011; Blanchard, 2004; Bogaert, 2006). At the genetic level, twin and genome-wide findings support a complex polygenic architecture with individually small effects rather than a single deterministic locus (Bailey et al., 2016; Ganna et al., 2019; Zietsch et al., 2021). These findings are compatible with a developmental-systems view in which genetic differences bias probabilities and constraints but do not specify adult orientation independently of developmental context (Oyama, 2000; Griffiths and Tabery, 2013).

The aim of this article is therefore integrative rather than mono-factorial: to align evidence with distinct but interacting explanatory questions and to evaluate whether an attraction-centered framework linking evolutionary maintenance, developmental assembly, motivational organization, and context-sensitive expression provides a useful account of persistence and variation in male and female same-sex orientations. The term motivational architecture denotes an emergent and progressively stabilized organization of attraction-related dispositions, not a fully preformed internal program. Likewise, canalization refers to relative stabilization of developmental trajectories, not insulation from environmental influence. Developmental plasticity and canalization can coexist, and their balance may differ among components of sexual orientation, between sexes, and across individuals (West-Eberhard, 2003; Fausto-Sterling, 2012).

This article is a conceptual analysis rather than a systematic review. The literature was selected through a targeted narrative strategy intended to represent the principal empirical and theoretical lines relevant to the framework, including supportive, mixed, and conflicting findings. The synthesis draws on established work in evolutionary, developmental, genetic, neurobiological, and socioecological research on sexual orientation, complemented by citation tracing and targeted searches of the relevant literature. The aim is not exhaustive coverage or meta-analytic weighting, but theoretically informative representation of the major competing interpretations and the evidence that bears on them.

## 2 Quantitative evidence, fecundity patterns, and genetic models

Quantitative analyses of fecundity and pedigree structure provide an important, but methodologically heterogeneous, source of evidence concerning the evolutionary maintenance of male androphilia. Studies differ substantially in the phenotype examined, the kin categories included, the demographic context, and the operational definition of the female fecundity effect (FFE). Some analyses measure only maternal reproductive output or sibship size; others examine fecundity across maternal and paternal aunts, grandmothers, or broader pedigrees. These designs do not test identical predictions, and their numerical estimates should therefore not be treated as interchangeable measures of a single effect.

A series of studies reported increased fecundity among female relatives in the maternal lineage of androphilic men, with little or no corresponding increase among homologous paternal-line relatives (Camperio Ciani et al., 2004, 2009, 2012; Iemmola and Camperio Ciani, 2009; Camperio Ciani and Pellizzari, 2012). These studies do not all derive from one repeatedly analyzed sample. They include multiple datasets with differing degrees of continuity: some analyses were partly cumulative with earlier samples, whereas others used newly collected samples to address distinct predictions, including bisexual probands, direct assessment of female relatives, and comparative maternal-versus-paternal non-parental kin. Their convergence therefore constitutes convergent evidence across multiple datasets within the same research program. At the same time, continuity of investigators and methodology, together with some partial sample overlap in selected analyses, means that this convergence should not be equated with fully independent external replication.

Independent studies have produced a more heterogeneous picture. Some report larger family size or partial maternal-line effects without directly testing the full pedigree-level prediction (King et al., 2005; Rahman et al., 2008; Schwartz et al., 2010). More recent large, preregistered, or population-based analyses have often failed to confirm a mother-based or maternal-line fecundity advantage and in some cases have observed paternal-line effects or no lineage asymmetry (Blanchard et al., 2020; Ablaza et al., 2022; Semenyna et al., 2023; Fořt et al., 2024, 2025).

These findings require a more differentiated evaluation than a simple supportive-versus-null classification. A study may be non-supportive for a mother-based FFE while not testing the extended maternal-line prediction, whereas a family-size study may be informative about overall pedigree structure but unable to discriminate X-linked sexually antagonistic selection from autosomal alternatives. Conversely, repeated maternal-line findings within one research program increase internal coherence but do not establish cross-population generality. The quantitative evidence is therefore best evaluated study by study, with explicit attention to phenotype, kin category, population structure, sample provenance, and the degree to which FBOE, sibship size, cohort, and ascertainment are controlled.

Table 1 summarizes representative empirical tests and reports study-level sample sizes, outcomes, effect estimates, interpretations, limitations, and research provenance. It is intended as a structured conceptual synthesis rather than a meta-analysis. The purpose is to make the evidential balance transparent while preserving the distinction between empirical observations and the evolutionary models proposed to explain them.

**TABLE 1:** Representative empirical tests of female fecundity, lineage effects, fraternal birth order, and X-linked sexually antagonistic selection in male androphilia.

References	Sample / population	Operational test	Representative quantitative result	Interpretation	Limitations / provenance
Camperio Ciani et al., 2004	98 homosexual + 100 heterosexual men; > 4,600 relatives; N Italy	Maternal-vs-paternal fecundity	Mothers 2.69 vs 2.32, *p* = 0.02; maternal aunts 1.98 vs 1.51, *p* = 0.001; maternal-grandparent descendants 2.17 vs 1.83, *p* = 0.001; paternal aunts NS.	Supports extended maternal-line asymmetry.	Small, retrospective, targeted; family-size correlation. Same program; foundational.
Iemmola and Camperio Ciani, 2009	250 men: 152 homosexual, 98 heterosexual; Italy	FFE; aunts; first-born test; paternal comparison	Mothers 2.73 vs 2.07, *p* = 0.001; first-born mothers 1.78 vs 1.54, *p* = 0.02; maternal aunts 1.96 vs 1.43, *p* = 0.001; paternal aunts NS; paternal uncles 1.75 vs 2.00, *p* = 0.05 (opposite).	Extended maternal-line pattern; first-born comparison argues against simple FBOE.	Retrospective; partly cumulative with 2004; not independent.
Camperio Ciani et al., 2009	239 men: 88 heterosexual, 86 bisexual, 65 homosexual	Maternal-line fecundity across orientations	Mothers 2.09/2.67/2.63, *p* = 0.01; mothers+maternal aunts 1.80/2.27/2.28, *p* = 0.001; paternal-line females *p* = 0.15.	Similar maternal signal in bisexual/homosexual families; older-brother excess only in homosexual probands.	Modest, retrospective; separate same-program sample.
Camperio Ciani et al., 2012	161 women: 61 maternal relatives of homosexual men, 100 controls	Direct maternal-relative fecundity	Mothers 2.51 vs 2.10, *p* = 0.01; maternal aunts 2.33 vs 1.47, *p* = 0.01; combined 2.48 vs 1.78, *p* < 0.001; complicated pregnancies 0.08 vs.25, *p* = 0.004.	Supports higher maternal female-relative fecundity.	Small; 12 exposed aunts; multiple comparisons. Same program.
Camperio Ciani and Pellizzari, 2012	264 homosexual + 240 heterosexual probands; ∼2,100 female relatives; Europe	Homologous maternal-vs-paternal female kin	Maternal aunts 1.96 vs 1.54, *F* = 21.28, *p* < 0.001; maternal grandmothers 3.67 vs 3.31, *F* = 3.97, *p* = 0.047; paternal aunts/grandmothers NS.	Direct within-program support for extended maternal-line asymmetry.	Retrospective; some overlap with earlier samples. Same program.
King et al., 2005	301 gay + 404 heterosexual White British men; STI clinic	Overall family-size proxy	Family-size OR = 1.02, 95% CI 1.01–1.03; adjusted OR = 1.02, 95% CI 1.00–1.03.	Larger family size does not isolate female-line fecundity.	Clinic sample; mixed-sex kin. Independent.
Rahman et al., 2008	147 homosexual + 155 heterosexual British men; White/non-White strata	Maternal female-relative fecundity	White maternal-aunt fecundity elevated; non-White stratum showed different/opposite kin patterns.	Mixed, population-dependent evidence.	Small heterogeneous non-White subgroup; erratum corrected counts, not tests. Independent.
Schwartz et al., 2010	694 homosexual + 894 heterosexual U.S. men	Family structure; secondary aunt reanalysis	Original study non-supportive. Secondary reanalysis: maternal aunts 1.532 vs paternal aunts 1.26, χ^2^ = 56.2, df = 1, *p* < 0.001.	Original non-supportive; reanalysis compatible but not independent replication.	Female fecundity not primary outcome; secondary assumptions. Independent original study.
Blanchard et al., 2020	14 samples/10 archived studies; *N* = 5,390	FBOE vs sibship-based FFE proxy	FBOE OR = 1.38, 95% CI 1.14–1.66, *p* < 0.001; FFE OR = 1.00, 95% CI.81–1.23, *p* = 1.00.	Supports FBOE, not tested sibship FFE; extended kin not tested.	Restricted family method; daughters excluded. Independent.
Ablaza et al., 2022	9,073,496 Dutch registers; 26,542 men + 33,534 women in same-sex unions	Joint sibship FBOE/FFE	Older-brother OR = 1.069 overall; 1.082 men; 1.055 women; no positive FFE.	Strong FBOE without positive sibship-based FFE.	Same-sex union outcome; registry/cohort effects. Independent.
Semenyna et al., 2023	1,061 Samoans: 703 gynephilic men, 358 fa’afafine	Joint FBOE + total-sibship FFE	FBOE OR = 1.19, 99.5% CI 1.03–1.39, *p* = 0.001; FFE OR = 1.03, 99.5% CI.93–1.14, *p* = 0.424.	FBOE persists; generalized sibship FFE absent; extended kin not tested.	Culturally specific, high-fertility setting. Independent.
Fořt et al., 2024	7,312 straight men, 459 gay men; 3,352 straight women, 79 lesbian women; Czechia	Parent/grandparent reproduction	Mothers 2.20 vs 2.15, τ = 0.00, *p* = 0.966; maternal grandmothers 2.74 vs 2.80, τ = −0.01, *p* = 0.966; paternal grandmothers 2.95 vs 2.80, τ = 0.03, *p* < 0.001.	Non-supportive for a universal maternal-line X-linked pattern.	Online self-report; no aunts/relative ages. Independent.
Fořt et al., 2025	843 straight men; 698 gay men in Methods/Table 1 (abstract: 693); 331 straight women; 265 lesbian women	Extended-kin reproduction	Mothers OR = 1.00, 95% CI.96–1.05, *p* = 0.920; maternal aunts OR = 0.99, 95% CI.91–1.08, *p* = 0.766; paternal grandparents OR = 1.09, 95% CI 1.03–1.16, *p* = 0.006.	Non-supportive maternal-line result; paternal-grandparent effect.	Self-report/missing kin; source N discrepancy. Independent.

FFE, female fecundity effect; FBOE, fraternal birth-order effect; SAS, sexually antagonistic selection; NS, not significant. The table is a structured conceptual synthesis rather than a meta-analysis. Mother- or sibship-based FFE measures are not equivalent to extended maternal-line tests using non-parental female kin. Representative estimates are transcribed from the cited publications; the Fořt et al. (2025) source contains an internal sample-size discrepancy (693 gay men in the abstract; 698 in corrected-proof Methods/Table 1).

### 2.1 Evidence for sexually antagonistic selection (SAS)

The specific X-linked sexually antagonistic selection (SAS) hypothesis considered here predicts more than increased reproduction by the mother of an androphilic male. SAS in general need not be X-linked; this submodel proposes that one or more X-linked liability factors may reduce direct reproduction in some male carriers while increasing reproductive output in female carriers. Because a male receives his single X chromosome from his mother, the critical prediction is preferentially elevated female fecundity in the maternal lineage, with little or no homologous paternal-line increase.

Camperio Ciani et al. (2004) provided the initial evidence. Among 98 homosexual and 100 heterosexual male probands comprising more than 4,600 relatives, mothers of homosexual men had 2.69 versus 2.32 offspring (*p* = 0.02) and maternal aunts 1.98 versus 1.51 (*p* = 0.001), whereas paternal aunts showed no corresponding increase. The study also reported higher cumulative reproductive output among sons and daughters of the maternal grandparents (2.17 vs. 1.83, *p* = 0.001), a measure combining mothers, maternal aunts, and maternal uncles.

Iemmola and Camperio Ciani (2009) examined an enlarged Italian dataset of 152 homosexual and 98 heterosexual men, partly cumulative with the earlier program. Mothers again showed higher fecundity (2.73 vs. 2.07, *p* = 0.001), as did maternal aunts (1.96 vs. 1.43, *p* = 0.001), while paternal-aunt fecundity did not differ significantly. Mothers whose homosexual son was first-born also showed higher fecundity than corresponding control mothers (1.78 vs. 1.54, *p* = 0.02), a comparison that cannot be generated by the proband having older brothers.

A separately collected sample including 88 heterosexual, 86 bisexual, and 65 homosexual men tested a distinct prediction (Camperio Ciani et al., 2009). Maternal fecundity was higher in bisexual and homosexual families: mothers had 2.09, 2.67, and 2.63 offspring, respectively (*p* = 0.01), and mothers plus maternal aunts 1.80, 2.27, and 2.28 (*p* = 0.001). Paternal-line female fecundity did not differ significantly, while an excess of older male siblings was restricted to homosexual, not bisexual, probands. This dissociation is relevant because it separates the older-brother pattern from the maternal-line fecundity signal.

Camperio Ciani et al. (2012) directly assessed 161 European maternal female relatives. Mothers of homosexual men had 2.51 versus 2.10 offspring (*p* = 0.01), maternal aunts 2.33 versus 1.47 (*p* = 0.01), and the combined comparison 2.48 versus 1.78 (*p* < 0.001). In the most direct maternal-versus-paternal test, Camperio Ciani and Pellizzari (2012) compared 264 homosexual and 240 heterosexual probands and approximately 2,100 female relatives. Maternal aunts had 1.96 versus 1.54 offspring (*F* = 21.28, *p* < 0.001) and maternal grandmothers 3.67 versus 3.31 (*F* = 3.97, *p* = 0.047), whereas paternal aunts (1.83 vs. 1.83, *p* = 0.960) and paternal grandmothers (3.42 vs. 3.47, *p* = 0.977) showed no increase.

These studies provide convergent evidence across multiple datasets within the same research program, not fully independent replications. Some samples overlap partly and investigators and methods recur, although other datasets were newly collected or addressed different kin classes and predictions. The pattern therefore strengthens the internal empirical basis of the X-linked SAS hypothesis while leaving external generality unresolved.

Independent evidence is mixed. King et al. (2005) found slightly larger family size among 301 gay than 404 heterosexual White British men (adjusted OR ≈ 1.02, 95% CI 1.00–1.03), but the combined kin outcome was not a lineage-specific female-fecundity test. Rahman et al. (2008) reported increased maternal-aunt fecundity in a White British subsample, whereas the non-White subsample showed a different pattern. Schwartz et al. (2010) did not interpret their data as predominant maternal transmission; a later reanalysis estimated maternal- versus paternal-aunt fecundity of 1.532 versus 1.26 (χ^2^ = 56.2, df = 1, *p* < 0.001), but this secondary analysis is not an independent replication (Camperio Ciani and Pellizzari, 2012).

Recent studies provide important non-supportive evidence. Fořt et al. (2024) found no maternal fecundity advantage in mothers or maternal grandmothers of gay men and a small paternal-grandmother increase. Fořt et al. (2025) again found no effect in mothers (OR = 1.00, 95% CI 0.96–1.05, *p* = 0.920) or maternal aunts, but a modest paternal-grandparent effect (OR = 1.09, 95% CI 1.03–1.16, *p* = 0.006). These findings are inconsistent with a simple universal X-linked maternal-line prediction.

A critical distinction concerns the relation between FFE and the fraternal birth-order effect (FBOE). Mother- or sibship-based FFE measures share the proband’s sibship structure and are therefore statistically entangled with FBOE. By contrast, maternal-aunt or maternal-grandmother fecundity is not mechanically determined by the proband’s number of older brothers and more directly tests an extended pedigree prediction. Extended-kin results remain vulnerable to familial, demographic, socioeconomic, cultural, recall, and ascertainment confounding.

A stringent future test should therefore compare mothers, maternal aunts, maternal grandmothers, and homologous paternal-line female relatives within the same pedigrees while modeling FBOE, sibship composition, cohort, population-level fecundity, familial clustering, and ascertainment. The evidence summarized in Table 1 neither establishes X-linked SAS as universal nor reduces it to maternal fecundity alone; it identifies a specific falsifiable prediction of extended maternal-line asymmetry.

### 2.2 Interpretation and modeling: polygenic liability, antagonistic pleiotropy, and X-linked SAS

The maternal-line asymmetry described in section “2.1 Evidence for sexually antagonistic selection (SAS)” is theoretically compatible with X-linked sexually antagonistic selection, but this specific pedigree model should be distinguished from the broader question of the genetic architecture of male androphilia. Evidence from twin, family, and genomic studies at present suggests that sexual orientation is partly heritable and highly polygenic, with many variants of individually small effect rather than a single deterministic locus. Failure of a particular X-linked model would therefore not imply absence of genetic liability; it would instead constrain one proposed mechanism by which part of that liability may be transmitted and maintained.

Earlier population-genetic modeling already pointed in this direction. Camperio Ciani et al. (2008) compared alternative one- and two-locus models under empirical constraints derived from the observed frequency of male homosexuality, reduced direct reproduction in homosexual men, increased fecundity in female maternal relatives, and the reported asymmetry between maternal and paternal lineages. Under those assumptions, simple one-locus formulations did not reproduce the full set of empirical patterns, whereas a two-locus sexually antagonistic model containing at least one X-linked component provided a substantially better fit. The relevance of this result lies less in the precise number of loci assumed in the original model than in the more general conclusion that stable persistence cannot be explained by a single-factor architecture and is more naturally represented by interacting genetic effects with sex-dependent fitness consequences.

Contemporary genomic evidence strengthens the polygenic component of this interpretation while broadening the possible location and action of the relevant variants. Large-scale genomic analyses indicate a highly polygenic architecture for same-sex sexual behavior and related phenotypes, with substantial heterogeneity and partly sex-differentiated effects (Ganna et al., 2019; Zietsch et al., 2021). Accordingly, any X-linked contribution is unlikely to constitute the entire genetic architecture. A more general formulation is that male androphilic liability may arise from many autosomal and sex-linked variants whose effects differ in magnitude, penetrance, sex specificity, developmental context, and pleiotropic consequences.

This broader framework is compatible with antagonistic pleiotropy. Zietsch et al. (2021) found that genetic effects associated with same-sex sexual behavior were also associated, among individuals reporting exclusively opposite-sex sexual behavior, with a greater number of opposite-sex sexual partners. Evolutionary simulations indicated that such a mating advantage could contribute to the persistence of alleles associated with same-sex behavior. This genome-wide result does not demonstrate the specific maternal-line mechanism proposed by X-linked SAS, but it supports the more general principle that variants associated with a reproductive cost in some phenotypic contexts may persist because they confer mating or reproductive advantages in other carriers or contexts.

The specific X-linked SAS hypothesis can therefore be viewed as a more restrictive submodel within a broader polygenic antagonistic framework. It makes an additional pedigree prediction: if some sexually antagonistic variants are X-linked and increase female reproductive success, enhanced fecundity should preferentially appear among female relatives in the maternal lineage rather than symmetrically across both parental lines. This prediction is stronger than the generic prediction of antagonistic pleiotropy and is correspondingly easier to falsify.

Phenotypic findings in putative female carriers are also compatible with this broader interpretation, although they remain preliminary. Camperio Ciani et al. (2012) reported that mothers and maternal aunts of homosexual men differed from comparison women not only in fecundity but also in reproductive health, extraversion, family stability, and attitudes toward romantic and social norms. The study was based on a relatively small sample and requires replication, but the pattern is compatible with the possibility that variants contributing to male androphilic liability may have broader behavioral or reproductive effects in females rather than influencing fecundity through a single physiological pathway.

This interpretation also helps explain why bisexual men are theoretically informative. In Camperio Ciani et al. (2009), maternal-line female fecundity was similarly elevated in bisexual and homosexual families, whereas an excess of older male siblings was restricted to homosexual probands. This dissociation is consistent with a dimensional liability related to androphilic motivation rather than with a categorical identity phenotype and further suggests that different genetic and developmental pathways may contribute to different points along the phenotypic distribution.

The relation between genetic liability and developmental mechanisms should therefore not be framed as an alternative. FBOE, maternal immune responses, prenatal endocrine conditions, and other developmental factors can contribute additional variance in phenotypic expression without themselves accounting for familial transmission. A polygenic liability may alter developmental probabilities, while nonshared prenatal or stochastic developmental processes determine whether and how that liability becomes expressed in a particular individual.

Accordingly, the present framework distinguishes three nested claims. First, male androphilia has a partly heritable, highly polygenic architecture. Second, antagonistic pleiotropy offers a plausible general mechanism by which some associated variants may persist despite reduced direct reproduction in a subset of male carriers. Third, X-linked SAS is a more specific hypothesis predicting a maternal-line pedigree asymmetry within that broader architecture. Repeated failure of the extended maternal-line prediction would weaken the third claim without eliminating the first two.

### 2.3 Female lesbian orientation: pedigree signature and balancing selection (BS)

Female lesbian orientation displays a pedigree and fecundity pattern that differs from the maternal-line asymmetry considered for male androphilia. Camperio Ciani et al. (2018) examined 1,458 adult women—487 lesbian, 163 bisexual, and 808 heterosexual respondents—and 30,203 relatives. Direct reproduction was markedly lower among lesbian women: 0.09 versus 0.33 offspring in the full sample (age-adjusted *B* = −0.292, *p* < 0.001) and 0.31 versus 1.54 among women aged 45 years or older (*B* = −1.16, *p* < 0.001).

Pedigree effects were heterogeneous rather than uniformly elevated (Table 2). Significant increases occurred in parents, paternal aunts, and maternal uncles, whereas maternal aunts, paternal uncles, and grandparents showed no significant increase. Lesbian respondents also reported more siblings and a higher prevalence of non-heterosexual female relatives in their pedigrees (2.83% vs. the 0.63% reference prevalence in heterosexual respondents’ pedigrees; χ^2^ = 105.34, df = 1, *p* < 0.001).

**TABLE 2:** Published quantitative patterns in families of lesbian women (Camperio Ciani et al., 2018).

Comparison / measure	Published quantitative result	Reported statistic	Interpretive relevance
Focal lesbian vs heterosexual women — full sample	0.09 vs 0.33 offspring	*B* = −0.292, *p* < 0.001	Marked reduction in direct reproduction of the focal phenotype.
Focal women aged ≥ 45 years	0.31 vs 1.54 offspring	*B* = −1.16, *p* < 0.001	Direct-reproduction difference persists near the end of the reproductive span.
Pedigree size	21.93 vs 20.53 relatives	Age-adjusted *p* = 0.053	Marginal family-structure difference; not a significant fecundity effect by itself.
Number of siblings	1.43 vs 1.22	*B* = 0.18, *p* = 0.002	Larger sibship among lesbian respondents; family-size context rather than kin fecundity.
Parents	2.43 vs 2.22 offspring	*B* = 0.174, *p* = 0.002	Significant family-level reproductive increase.
Paternal aunts	1.99 vs 1.81 offspring	*B* = 0.15, *p* = 0.009	Significant paternal-side increase.
Maternal uncles	1.81 vs 1.55 offspring	*B* = 0.25, *p* < 0.001	Significant maternal-side increase in a male kin class.
Paternal uncles	1.81 vs 1.68 offspring	*B* = 0.10, *p* = 0.075	Non-significant; compensation is not uniform across paternal kin.
Maternal aunts	1.79 vs 1.84 offspring	*B* = −0.05, *p* = 0.934	Null result; no uniform maternal-side increase.
Grandparents	Paternal 3.47 vs 3.42; maternal 3.52 vs 3.46 offspring	Overall *B* = 0.067, *p* = 0.266	No significant overall grandparent effect.
Non-heterosexual female relatives in pedigrees	2.83% vs 0.63% reference prevalence in heterosexual respondents’ pedigrees	χ^2^ = 105.34, df = 1, *p* < 0.001	Familial aggregation; distinct from the fecundity test itself.

Values are published results reported by Camperio Ciani et al. (2018); no new statistical analysis is performed in the present Conceptual Analysis. NS, not significant. The heterogeneous pattern—significant effects in selected maternal and paternal kin classes together with several null comparisons—should not be described as a uniform fecundity increase across the pedigree. The balancing-selection interpretation remains model-compatible and requires independent replication.

The bilateral structure of these pedigree effects is theoretically important. Unlike the specific X-linked SAS prediction considered for male androphilia, the compensatory pattern was not restricted to the maternal female line: significant increases occurred in selected kin classes on both paternal and maternal sides. The several null kin comparisons caution against describing the effect as a uniform pedigree-wide increase. Together with the reduced direct reproduction of lesbian women, this pattern is compatible with a balancing-selection interpretation in which fitness costs in a low-reproduction minority phenotype may be offset partly by increased reproduction elsewhere in the pedigree. The interpretation remains model-compatible and requires independent replication.

The principal evidence derives from one large pedigree study within the same research program, and independent pedigree-level replication is still lacking. The observed bilateral compensation is therefore informative because it differs qualitatively from the maternal-line pattern proposed for male androphilia, but its generality across populations remains unknown. A stronger test would require independent samples in which direct reproduction, sexual orientation, and the fecundity of homologous maternal and paternal kin classes are measured within the same pedigrees and adjusted for cohort, family size, demographic context, and ascertainment.

Accordingly, the present analysis treats balancing selection as one plausible maintenance model for stable lesbian orientation, not as a unique explanation. Failure to reproduce bilateral kin-level compensation in independent populations would substantially weaken this interpretation.

Representative published quantitative results and their balancing-selection interpretation are summarized in Table 2.

### 2.4 Female gynephilia as a motivational trait: implicit measures, plasticity, and alternative functional hypotheses

The kin-level pattern described above concerns relatively stable lesbian orientation and should not be generalized automatically to female gynephilia as a broader motivational trait. A large cross-cultural study using implicit and explicit measures reported that gynephilic responses were widespread among women, frequently coexisted with attraction to men, were only weakly coupled to non-heterosexual identity, and showed no distinctive fecundity signature associated with gynephilia itself (Camperio Ciani and Colledani, 2025). These findings are more consistent with a broadly distributed and developmentally plastic motivational dimension than with a discrete low-frequency reproductive phenotype.

This distinction is important because evolutionary hypotheses proposed for female sexual fluidity do not necessarily address the same phenomenon as models proposed for stable lesbian orientation. Kuhle and Radtke (2013) proposed an alloparenting hypothesis in which female sexual fluidity may have facilitated pair-bond formation with unrelated female alloparents under conditions in which paternal investment was absent, reduced, or unreliable. In this account, flexible same-sex attraction or bonding could have increased access to allocare and resources under particular ancestral circumstances. The proposal is theoretically specific but remains only indirectly tested; demonstrating an association between sexual fluidity and actual alloparental assistance or reproductive benefit under the predicted ecological conditions would provide a stronger test.

Kanazawa (2017) proposed a different functional account in which female sexual fluidity may have reduced conflict among co-wives in polygynous marriages. Analyses of Add Health data reported in that study found that women, but not men, showing greater sexual fluidity had more children and that early marriage or parenthood predicted subsequent increases in female sexual fluidity. These correlations are consistent with some predictions of the hypothesis, but they do not directly test the proposed causal mechanism linking sexual relationships among co-wives to reduced conflict, greater cooperation, and enhanced reproductive success. The critical adaptive pathway therefore remains incompletely demonstrated. This distinction is also consistent with the caution expressed in Camperio Ciani and Colledani (2025): evidence for female sexual fluidity should not by itself be treated as evidence for the specific fitness mechanism proposed to explain its evolutionary maintenance.

The alloparenting and co-wife hypotheses thus offer competing or potentially complementary functional interpretations of female sexual fluidity, but neither is equivalent to the bilateral balancing-selection model considered for stable lesbian orientation. The former hypotheses concern possible context-dependent benefits of flexible female attraction or bonding, whereas the balancing-selection interpretation concerns a family-level reproductive signature associated with a more stable minority orientation. Evidence for one class of prediction would not, by itself, validate the other.

The resulting picture is therefore layered rather than unitary. Gynephilic motivation may be relatively widespread and variably expressed; sexual fluidity may alter the coupling between attraction, behavior, and identity; and a smaller subset of women may develop a more stable lesbian orientation associated with substantially reduced direct reproduction. Whether these phenomena share a common evolutionary history, reflect partially distinct developmental pathways, or arise from several maintenance processes remains unresolved. Current evidence is insufficient to privilege a single adaptive account of female gynephilia.

## 3 Alternative evolutionary models and reconciliation

Alternative evolutionary models should be compared at the level of the specific predictions they make rather than treated as mutually exclusive explanations of same-sex orientation as a single phenotype. Genetic architecture, evolutionary maintenance, developmental expression, and socially mediated behavior are distinct questions.

For male androphilia, polygenic liability is compatible with several maintenance processes. Antagonistic pleiotropy is the broadest formulation: variants may persist if reproductive costs in some carriers are offset by mating or reproductive advantages in others. The genomic findings of Zietsch et al. (2021) are consistent with this general mechanism. The specific X-linked SAS hypothesis adds the narrower prediction that a subset of such effects generates preferential maternal-line female fecundity.

Overdominance represents a different mechanism in which heterozygotes have higher fitness than either homozygote. Earlier analyses found that simple overdominance formulations did not reproduce the combination of low-frequency persistence and reported maternal-line asymmetry as successfully as multilocus sexually antagonistic models under the empirical assumptions then available (Gavrilets and Rice, 2006; Camperio Ciani et al., 2008). This does not refute all possible overdominance models; it indicates that simple autosomal heterozygote advantage does not naturally predict lineage-specific pedigree structure.

Genomic imprinting could in principle generate parent-of-origin effects, but available models and pedigree data have not produced a consistent grandparent-of-origin pattern uniquely supporting imprinting. More generally, contemporary polygenic evidence allows heterogeneous mechanisms across loci: autosomal and sex-linked variants may have antagonistic, balancing, nearly neutral, or other effects. Population-level persistence need not therefore reflect one mechanism acting uniformly across the genome.

The same distinction applies to female same-sex orientation. Bilateral pedigree compensation in stable lesbian orientation is compatible with balancing selection but requires independent replication; failure of that signature would weaken the maintenance model without implying absence of heritable liability. Functional hypotheses for female sexual fluidity, such as alloparenting or co-wife cooperation, concern possible context-dependent benefits and operate at a different explanatory level.

The resulting synthesis is hierarchical: polygenic liability concerns predisposition; antagonistic pleiotropy, balancing selection, overdominance, and parent-of-origin mechanisms concern possible evolutionary maintenance; and FBOE, maternal immune effects, endocrine differentiation, stochasticity, and socioecological experience influence phenotypic realization. Models become direct competitors only when they make incompatible predictions about the same phenotype and level. Contrasting pedigree-level predictions are summarized in Table 3.

**TABLE 3:** Schematic pedigree-level predictions of alternative evolutionary models.

Model	Target	Male prediction	Female prediction	Key pedigree test
SAS	Male androphilia; female-carrier fecundity	Reduced direct reproduction in a subset of androphilic males	Increased fecundity in female carriers	Preferential maternal-line female-kin fecundity increase under the specific X-linked submodel, with little/no homologous paternal-line increase.
BS	Stable female lesbian orientation	—	Reduced direct reproduction in focal lesbian women with increased reproduction among heterosexual kin	Bilateral kin-level compensation across maternal and paternal sides.
OD	Heterozygote advantage	Benefits may appear in both sexes	Female-kin fecundity advantage may occur on both lines	Maternal and paternal fecundity increases expected under a simple autosomal formulation.
GI	Parent-of-origin effects	Depends on imprinting direction	Cross-generational asymmetries	Parent-of-origin / grandparental-line asymmetries.
Socioecological pathway	Context-dependent expression and fitness	Fitness costs may vary by social context	Expression and reproduction may vary by institutions and mating ecology	No single invariant pedigree signature; population/context dependence predicted.

SAS, sexually antagonistic selection; BS, balancing selection; OD, overdominance; GI, genomic imprinting. This table is heuristic. Models compete only where they make incompatible predictions about the same explanatory level.

## 4 Reconciliation across levels

The explanatory levels distinguished here are analytical tools, not a unidirectional causal hierarchy. Sexually antagonistic selection and balancing selection concern evolutionary maintenance, whereas prenatal endocrine variation, maternal immune effects, neural differentiation, learning, and socioecological experience concern developmental and proximate processes. Developmental-systems and reciprocal-causation perspectives caution against treating these as independent domains: development produces the phenotypic variation on which selection acts, while evolutionary history shapes inherited and developmental constraints (Laland et al., 2011; Oyama, 2000; Griffiths and Tabery, 2013; West-Eberhard, 2003).

Genetic variation is therefore treated as probabilistic constraint and bias rather than as a fixed program. Twin studies indicate heritable contributions in both sexes, and genome-wide analyses support a highly polygenic architecture with many small and partly sex-differentiated effects (Kendler et al., 2000; Långström et al., 2010; Sanders et al., 2015, 2017; Ganna et al., 2019; Zietsch et al., 2021). Earlier Xq28 linkage findings are not equivalent to a single-locus account, and broad GWAS phenotypes such as ever having had a same-sex partner combine attraction, behavior, opportunity, and social context (Hamer et al., 1993; Sanders et al., 2015; Ganna et al., 2019).

Motivational architecture likewise denotes an emergent organization of attraction-related dispositions assembled through interactions among inherited variation, prenatal conditions, neural and endocrine development, organismic activity, social learning, and relationship experience. Biological and environmental factors acquire their effects through developmental systems rather than contributing as fully separable causes (Oyama, 2000; Griffiths and Tabery, 2013; Fausto-Sterling, 2012; Fausto-Sterling et al., 2012). Canalization therefore means relative stabilization, not causal closure; plasticity and stability can coexist at different levels of attraction, behavior, identity, and social expression.

Large internet-based implicit-association studies illustrate why these phenotypes should not be collapsed. In men, latent androphilic responses appear more broadly distributed than explicit gay or bisexual identification; in women, implicit gynephilic responses are widespread and weakly coupled to explicit non-heterosexual identity or consistent fecundity signatures (Colledani and Camperio Ciani, 2021; Camperio Ciani and Colledani, 2025). These self-selected samples are not prevalence estimates, but they underscore the distinction among motivation, orientation, behavior, and identity.

Figure 1 summarizes this reciprocal architecture. Adjacent levels are linked bidirectionally; within-individual experience can feed back from socioecological embedding toward ongoing developmental assembly; and reproductive, mating, and kin-directed outcomes feed back across generations to population-level evolutionary dynamics. The figure is therefore a heuristic map of interacting levels rather than a causal cascade.

**FIGURE 1:**
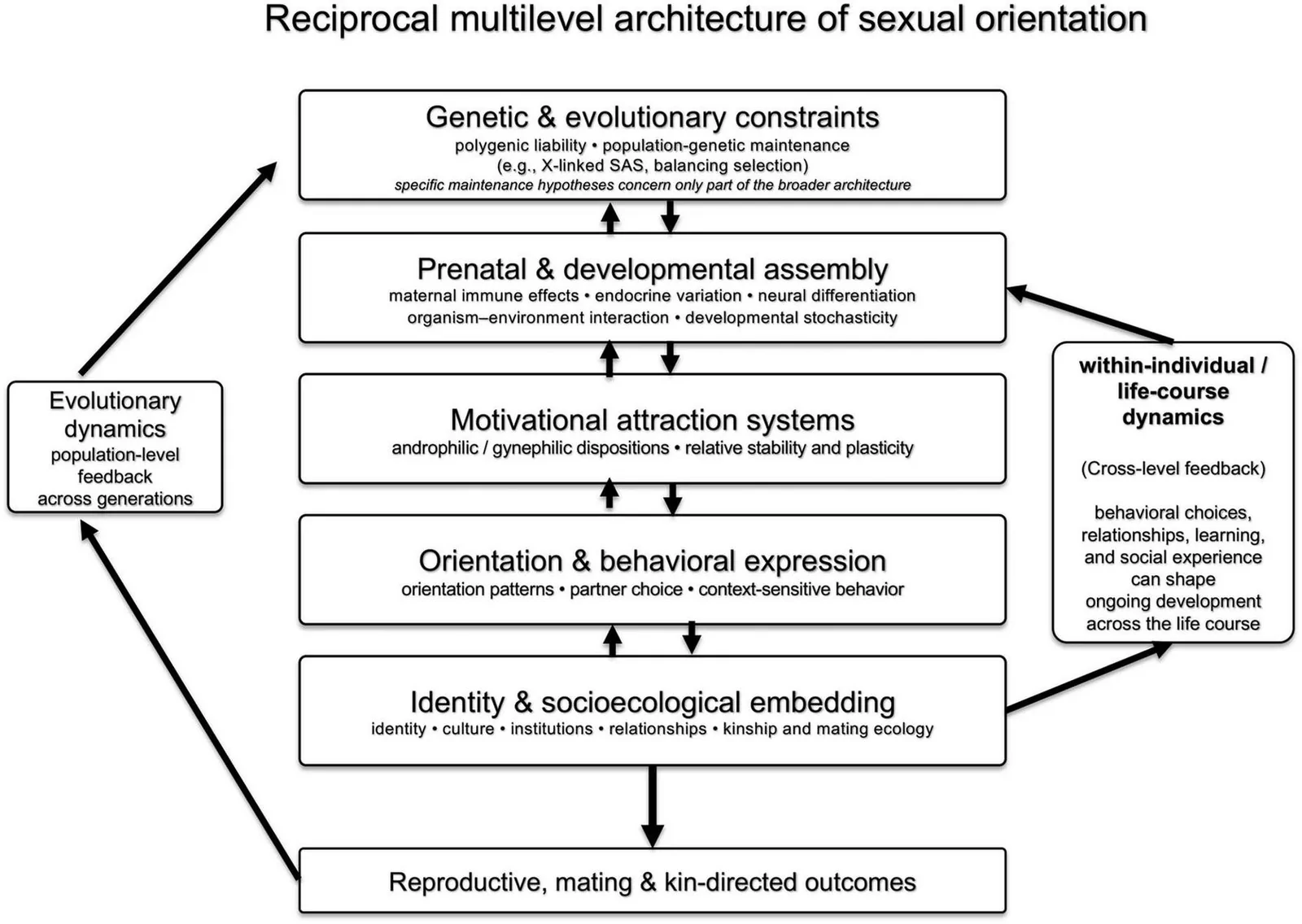
Reciprocal multilevel architecture of sexual orientation. Genetic and evolutionary constraints, prenatal and developmental assembly, motivational attraction systems, orientation and behavioral expression, and identity and socioecological embedding are analytically distinct but reciprocally connected. Bidirectional arrows indicate mutual influences between adjacent levels, whereas longer arrows represent cross-level feedbacks. Reproductive, mating, and kin-directed outcomes contribute to evolutionary dynamics across generations rather than feeding back genetically within individuals. The framework is heuristic and phenotype-specific: particular maintenance hypotheses, including X-linked SAS and balancing selection, concern only part of the broader polygenic, developmental, motivational, and socioecological architecture.

## 5 Prenatal environment and neurobiological pathways

Evolutionary maintenance models and developmental mechanisms address different aspects of the same phenotype, but they should not be treated as statistically or causally independent by assumption. Genetic variation, prenatal endocrine conditions, maternal immune processes, neural development, organismic activity, and later socioecological experience can interact across development. The distinction between ultimate and proximate explanation is therefore used as an analytical heuristic rather than as a claim that evolutionary and developmental processes form separate causal domains.

The fraternal birth-order effect (FBOE) is among the most replicated developmental correlates of male androphilia. Meta-analytic work has estimated an increase in the odds of male homosexuality with each additional older brother, although the magnitude depends on study design and family structure (Blanchard, 2018). The leading proximate account is the maternal immune hypothesis, according to which successive male pregnancies may alter maternal immune responses to male-specific antigens, potentially affecting later male neurodevelopment (Blanchard, 2001, 2004; Bogaert, 2006; Bogaert and Skorska, 2011).

Recent work has clarified that FBOE and female-fecundity effects must be estimated jointly when both are derived from the proband’s maternal sibship. Blanchard et al. (2020), analyzing 14 archived samples comprising 5,390 men, estimated approximately a 38% increase in the odds of homosexuality when comparing zero with one older brother, while their sibship-based FFE proxy showed no corresponding increase. Ablaza et al. (2022), using linked Dutch population-register data on more than nine million individuals, likewise found a robust older-brother association with later same-sex union but no positive FFE in joint multivariable models. Semenyna et al. (2023), in 1,061 Samoan participants, found that the FBOE remained detectable in a joint model (OR = 1.19, 99.5% CI 1.03–1.39, *p* = 0.001), whereas the total-sibship/FFE parameter did not (OR = 1.03, 99.5% CI 0.93–1.14, *p* = 0.424).

These findings require qualification of any simple claim that FBOE and FFE are merely complementary. At the conceptual level, a maternal immune pathway and an evolutionary maintenance mechanism can coexist because developmental causation and evolutionary persistence address different questions. At the empirical level, however, mother-based FFE and FBOE share the same sibship structure and must be disentangled statistically. Failure to detect a mother- or sibship-based FFE after controlling FBOE is therefore evidence against that specific fecundity prediction.

As reviewed in section “2.1 Evidence for sexually antagonistic selection (SAS),” the extended pedigree prediction considered under X-linked SAS is broader than mother-based FFE. Reported maternal-aunt and maternal-grandmother effects are less directly vulnerable to FBOE ascertainment because the reproductive output of these women is not mechanically determined by the proband’s own number of older brothers, and these women need not themselves have an androphilic son. This distinction does not make extended-kin data confound-free: reproductive output can cluster within families for genetic, demographic, socioeconomic, or cultural reasons. The most informative future designs should therefore estimate FBOE and extended pedigree effects within the same families, comparing maternal and paternal female kin while modeling sibship composition, familial fecundity, cohort, background population fecundity, and ascertainment.

Prenatal endocrine pathways constitute another component of developmental assembly. Experimental work in non-human mammals demonstrates that prenatal and perinatal hormones influence sex-differentiated neural development and partner-directed behavior, while human evidence from natural experiments and clinical populations indicates probabilistic associations between prenatal androgen exposure, gender-typed behavior, and later sexual orientation (Hines, 2004, 2011; Balthazart, 2011). These associations should not be interpreted as deterministic or as the unfolding of a fixed genetic program. They are better understood as developmental processes in which biological predispositions interact with other organismic and environmental influences over time.

Neurobiological findings can be situated similarly. Studies of sexually dimorphic brain structures and neural responses have reported group differences associated with sexual orientation (LeVay, 1991; Bao and Swaab, 2011), but causal direction, developmental timing, sample size, and the effects of experience remain important limitations. Such findings are therefore most informative when treated as correlates of developmental differentiation rather than direct readouts of an underlying evolutionary mechanism.

In females, developmental plasticity appears greater on average, with gynephilic attraction more weakly coupled to categorical identity and more variable across relationships and life stages (Diamond, 2003, 2008; Chivers, 2017). Prenatal endocrine influences may contribute to this distribution, but they operate within a developmental system that remains open to postnatal and socioecological modulation. This broader view is consistent with the framework developed here: evolutionary models concern the persistence of liability distributions, whereas developmental processes assemble phenotypes through reciprocal interactions rather than simply transmitting a preformed motivational program.

## 6 Child development and gender nonconformity

Childhood gender nonconformity is one of the most consistent developmental correlates of later non-heterosexual orientation, particularly in males (Bailey and Zucker, 1995; Rieger et al., 2008). Boys who show marked preferences for cross-gender play, toys, dress, peer groups, speech, or gestures are, on average, substantially more likely than gender-conforming boys to report homosexual or bisexual attractions in adolescence or adulthood. In girls the association is also present but generally weaker: childhood gender-nonconforming girls have elevated probabilities of later lesbian or bisexual attraction or identity, while many nevertheless develop heterosexual orientations or identities (Rieger et al., 2008; Rieger and Savin-Williams, 2012).

These associations should be interpreted developmentally rather than as direct behavioral readouts of a fixed latent orientation. Childhood gender nonconformity may reflect partially shared biological and developmental processes with later sexual orientation, but it is neither necessary nor sufficient for a particular adult outcome. Heritable variation contributes to childhood gender-typed behavior, yet heritability does not imply developmental immutability or identify a single causal pathway. Genetic, endocrine, neural, family, peer, and broader cultural influences can become mutually related over time through developmental processes (Oyama, 2000; Griffiths and Tabery, 2013; Fausto-Sterling, 2012; Fausto-Sterling et al., 2012).

From this perspective, early gendered behavior is best treated as one component of developmental assembly. Early tendencies can alter the social environments individuals encounter, the activities they select, and the responses they receive from others; those environments can in turn influence later behavior, self-concept, opportunity, and the salience or expression of attraction. This reciprocal view does not imply that social experience freely determines sexual orientation. Rather, it replaces a simple predisposition-versus-environment dichotomy with probabilistic developmental trajectories in which continuity and transformation are both possible (West-Eberhard, 2003).

Sex differences in average developmental stability remain relevant. In males, retrospective and longitudinal evidence suggests comparatively strong continuity between pronounced childhood gender nonconformity and later androphilic orientation, and adult sexual attraction is generally resistant to deliberate attempts at redirection (Bailey et al., 2016; Rieger and Savin-Williams, 2012). In females, the relation between childhood gendered behavior and adult attraction is less tightly coupled, while changes in the intensity or direction of attractions, partner sex, or identity labels are reported more frequently across relationships and life stages (Diamond, 2003, 2008; Chivers, 2017). This difference is better described as a difference in average developmental plasticity and coupling among components than as a contrast between biologically determined male orientation and socially determined female orientation.

Accordingly, canalization refers here to increasing stability of an attractional trajectory, not to closure of the developmental system. A relatively canalized adult orientation may still have emerged through reciprocal interactions among inherited dispositions, prenatal conditions, childhood behavior, embodied experience, and social context. Conversely, greater plasticity does not imply absence of biological predispositions. The relevant empirical question is how strongly different components—motivation, attraction, behavior, and identity—remain coupled or become dissociated across development.

Gender identity and sexual orientation are analytically distinct constructs. Developmental research on sex/gender diversity may inform general models of organism–environment interaction, but transgender and non-binary identities are not forms of sexual orientation and are therefore outside the principal explanatory target of the present analysis. This distinction is also relevant to culturally recognized gender categories discussed later: such roles should not be treated as interchangeable with Western sexual-orientation identities.

## 7 Cultural, socioecological context, and identity

Cross-cultural variation provides an important test of how socioecological conditions shape the expression and fitness consequences of same-sex orientation. Culturally recognized roles, kinship systems, mating institutions, stigma, and opportunities for reproduction can influence behavior, identity, family investment, and realized fecundity. Such variation does not necessarily identify the evolutionary mechanism responsible for the persistence of same-sex attraction, but it can alter how underlying motivational and developmental dispositions are expressed within particular populations.

The Samoan fa’afafine provide one of the best documented examples. Fa’afafine are feminine androphilic males occupying a culturally recognized gender category and have repeatedly been reported to show elevated avuncular tendencies, particularly toward nieces and nephews (Vasey and VanderLaan, 2010; VanderLaan and Vasey, 2011; VanderLaan et al., 2012, 2017). These findings demonstrate that a culturally structured role can channel male androphilia into distinctive patterns of kin-directed behavior. They are therefore important for understanding the interaction between sexual orientation, gendered social organization, and family investment.

The evolutionary interpretation of this pattern should nevertheless remain limited. Elevated avuncularity in Samoa does not by itself establish kin selection as a general maintenance mechanism for male androphilia across human populations. Its fitness consequences depend on the magnitude of the benefits transferred to kin, relatedness, demographic conditions, and the extent to which such investment compensates for reduced direct reproduction. More recent Samoan evidence further illustrates the need to distinguish social behavior from developmental and reproductive mechanisms. Semenyna et al. (2023), analyzing fraternal birth order and female fecundity jointly, found support for the fraternal birth-order effect but not for the previously proposed female-fecundity effect. Population-specific socioecological patterns therefore need not map onto a single evolutionary maintenance process.

A useful comparison is provided by the Urak-Lawoi of Ko Lipe, Thailand. Among this population, locally recognized feminine androphilic males are referred to as na-ning. Earlier research used the broader Thai term kathoey, but subsequent ethnographic clarification identified na-ning as the more precise local category; both Urak-Lawoi na-ning and Thai kathoey may be present on Ko Lipe (Camperio Ciani et al., 2016a; Vasey et al., 2016). The term na-ning is therefore used here as the population-specific category, without treating it as equivalent to Western sexual-orientation or gender-identity labels.

In the Urak-Lawoi sample, androphilic na-ning did not show greater avuncularity or kin-directed altruism than gynephilic men. Instead, higher levels of kin-directed and non-kin-directed altruism characterized the Urak-Lawoi population more generally and were associated with broader societal norms rather than sexual orientation specifically (Camperio Ciani et al., 2016a). This comparison subsequently generated a methodological debate. Vasey et al. (2016) questioned both the comparability of Ko Lipe with Samoa and aspects of the statistical analysis, whereas Camperio Ciani et al. (2016b) argued that participants had developed within a substantially traditional Urak-Lawoi social environment and that relevant confounders had been addressed through comparative tests and multivariate regression analyses. Regardless of this disagreement, the Urak-Lawoi findings did not reproduce the strong orientation-specific avuncular pattern reported in Samoa.

The contrast between Samoa and the Urak-Lawoi is therefore more informative as evidence for socioecological heterogeneity than as a decisive test between evolutionary theories. Comparable forms of male androphilia can become embedded in different cultural institutions, and those institutions may produce different patterns of kin investment, partnering, social identity, and reproductive opportunity. The absence of a uniform avuncular pattern across populations argues against treating one culturally specific social pathway as a universal explanation for the persistence of male androphilia.

More generally, culturally recognized categories such as fa’afafine or na-ning should not be treated as direct equivalents of Western sexual-orientation or gender-identity categories. They combine locally specific configurations of gender expression, social role, sexual attraction, and identity. Their comparative value lies precisely in showing that the mapping among motivational orientation, behavior, gendered role, and social identity is culturally variable.

These observations also reinforce the reciprocal developmental framework adopted in this article. Socioecological context does not operate only after sexual orientation has been formed. Cultural institutions can influence developmental experience, partnering opportunities, identity formation, family responses, kin-directed investment, and realized reproduction. These processes can in turn alter the phenotypic and fitness patterns on which evolutionary interpretations are based.

Cross-cultural evidence therefore supports a plural and population-sensitive interpretation. Socioecological institutions can modify the expression, social consequences, and realized fitness effects of same-sex orientation, but current evidence does not support treating any single culturally mediated pathway as a universal explanation of its evolutionary persistence. The principal value of comparative cases is instead to identify how different developmental and social environments interact with underlying motivational dispositions and thereby generate population-specific patterns of behavior and reproduction.

## 8 Discussion

The evidence reviewed here supports a multilevel account in which sexual orientation reflects a partly heritable, highly polygenic architecture interacting with developmental, motivational, and socioecological processes. The continuity between early pedigree work and contemporary genomic evidence lies not in confirmation of a particular locus or transmission pattern, but in recognizing that variation in orientation is compatible with complex heritable liability whose evolutionary persistence requires explanation (Camperio Ciani et al., 2004; Ganna et al., 2019; Zietsch et al., 2021).

The framework therefore separates genetic architecture from evolutionary maintenance. Polygenic liability can bias developmental probabilities without specifying a fixed adult outcome, while evolutionary mechanisms concern why associated variants persist. Antagonistic pleiotropy provides a broad maintenance principle, and the specific X-linked SAS hypothesis is a narrower pedigree-generating submodel predicting preferential maternal-line female fecundity. Repeated failure of that extended maternal-line signature would weaken the X-linked submodel without negating polygenic heritability or other antagonistic effects.

Evidence for the X-linked prediction remains mixed. Findings from one research program show repeated maternal-line fecundity effects, including in non-parental female relatives, but some samples overlap and external replication is limited. Independent studies vary by phenotype, kin category, population, and analytic design. Joint FBOE/FFE analyses show that an older-brother effect can persist when mother- or sibship-based fecundity effects disappear (Blanchard et al., 2020; Ablaza et al., 2022; Semenyna et al., 2023), while recent extended-kin studies provide direct non-supportive evidence for a universal maternal-line pattern (Fořt et al., 2024, 2025). The decisive test is therefore not maternal fecundity alone but an extended maternal-versus-paternal pedigree comparison controlling FBOE, sibship, cohort, background fertility, and ascertainment.

Contemporary genomic evidence broadens the evolutionary interpretation. Same-sex behavior is highly polygenic, and Zietsch et al. (2021) reported that associated genetic effects also predicted more opposite-sex partners among exclusively heterosexual individuals. This does not demonstrate X-linkage, but it supports antagonistic pleiotropy as a plausible general principle. Earlier multilocus modeling and current genomic results can thus be connected without treating the historical two-locus model as a literal description of genomic architecture.

For female orientation, the same distinction between architecture and maintenance is essential. Stable lesbian orientation, broader gynephilic motivation, bisexuality, sexual fluidity, behavior, and identity are not interchangeable phenotypes. The bilateral pedigree compensation reported for stable lesbian orientation is compatible with balancing selection but currently depends heavily on one research program and requires independent replication. Alloparenting and co-wife hypotheses instead address possible context-dependent functions of female sexual fluidity; evidence for such functions would not establish the pedigree-level balancing model, and vice versa.

Developmental mechanisms are likewise complementary to, rather than substitutes for, genetic liability. FBOE, maternal immune processes, prenatal endocrine variation, neural differentiation, childhood behavior, stochasticity, and continuing social experience can contribute to phenotypic realization. Motivational architecture denotes the emergent organization of attraction-related dispositions within this developmental system. Canalization refers to relative stabilization, not closure, and greater average female fluidity should not be interpreted as absence of biological predisposition.

Cross-cultural evidence further shows that socioecological context can alter the mapping from motivation to behavior, identity, kin investment, and realized fitness. The contrast between Samoan fa’afafine and Urak-Lawoi na-ning is therefore more informative about population-specific social organization than about a universal kin-selection mechanism. Culturally specific categories should not be equated automatically with Western orientation or gender-identity categories.

Several limitations remain. This is a conceptual analysis rather than a systematic review or meta-analysis and does not formally weight heterogeneous evidence. Positive pedigree findings are concentrated in one research program, some samples partly overlap, phenotype definitions vary, and demographic regimes differ. Conversely, some large independent studies use broad behavioral outcomes or kin measures that do not test the same pedigree prediction. The framework should therefore be judged by whether its specific predictions are reproduced independently across populations and methods.

The strongest future studies should integrate clearly defined attraction, orientation, behavior, and identity phenotypes with genomic, pedigree, developmental, and socioecological measures in the same populations. For male androphilia, priorities are joint tests of extended maternal-line asymmetry and FBOE and genomic tests linking antagonistic effects to realized fitness. For female orientation, independent tests of bilateral pedigree compensation should be separated from tests of gynephilic motivation and sexual fluidity. Across both sexes, the central question is which components of liability map onto which developmental pathways and fitness consequences.

The principal conclusion is therefore not that one evolutionary mechanism explains human same-sex orientation. The evidence is more consistent with a plural, phenotype-specific framework in which polygenic liability, developmental processes, motivational organization, and socioecological context interact reciprocally. X-linked SAS remains a specific falsifiable hypothesis for part of male androphilic liability; balancing selection remains a model-compatible but unreplicated interpretation of the reported lesbian pedigree pattern; and broader antagonistic pleiotropy provides a plausible bridge between earlier multilocus models and contemporary genomics.

## 9 Explicit testable predictions

The reciprocal multilevel framework is intended to generate discriminating predictions rather than a single omnibus test of whether sexual orientation is genetic, developmental, or cultural. Each test should specify the phenotype, explanatory level, and observations that would weaken the hypothesis under examination.

1. Specific X-linked SAS prediction. Enhanced reproductive output should preferentially occur among maternal-line female relatives of androphilic men, including aunts and grandmothers, rather than homologous paternal-line relatives. The asymmetry should survive control of FBOE, sibship composition, cohort, background fecundity, demographic context, and ascertainment; repeated independent failure would weaken the X-linked SAS hypothesis.

2. Polygenic antagonistic-pleiotropy prediction. Genetic effects associated with same-sex attraction or behavior should also predict mating or reproductive advantages in other phenotypic contexts, including exclusively heterosexual carriers, without requiring X-linkage or maternal-line asymmetry. Genomic studies should test sex- and phenotype-specific associations with mating success, reproduction, and pedigree-level fitness.

3. Bilateral balancing-selection prediction for stable lesbian orientation. Reduced direct reproduction among lesbian women should be accompanied by reproducible increases in heterosexual relatives on both maternal and paternal sides. Failure to reproduce bilateral compensation after adjustment for cohort, family size, demography, and ascertainment would weaken this maintenance model.

4. Functional predictions for female sexual fluidity. Alloparenting and co-wife hypotheses require evidence for their proposed mediators. Fluidity should predict allocare or cooperative support under the relevant ecological conditions in the former case, and reduced conflict or increased cooperation among co-wives in the latter; these pathways should in turn relate to reproductive or offspring outcomes.

5. Genetic-developmental integration prediction. FBOE, maternal immune processes, and related developmental factors should explain a subset of variance without eliminating familial or polygenic effects. Where genomic liability, sibship, gestational history, and biomarkers are measured jointly, developmental predictors should show additive or interactive associations rather than require a mutually exclusive genetic-versus-developmental model.

6. Motivational-architecture prediction. Developmental and biological predictors should relate more directly to measures of androphilic or gynephilic attraction than to culturally contingent identity labels alone. Attraction-based measures should show greater cross-context continuity, while behavior and identity should vary additionally with opportunity, norms, relationships, and institutions.

7. Reciprocal socioecological prediction. Comparable motivational dispositions should produce different behavioral, identity, kin-investment, and reproductive outcomes across cultural settings. Longitudinal and cross-cultural studies should test whether social roles, stigma, mating systems, kinship structures, and partnering opportunities modify the coupling among motivation, behavior, identity, and realized fitness.

8. Demographic-contingency prediction. If compensatory reproductive advantages contribute to maintenance, their magnitude should vary with demographic regime. Tests of X-linked SAS and female balancing selection should therefore model cohort and population-level reproductive conditions, with the expected direction and magnitude derived from the specific population-genetic model rather than assumed in advance.

Together, these predictions separate broad evidence for polygenic liability from narrower hypotheses about evolutionary maintenance and phenotypic expression. The framework gains support through convergence across genomic, pedigree, developmental, motivational, and socioecological measures within the same populations, and is weakened where its specific predictions repeatedly fail in adequately powered independent tests.
